# 

**Published:** 1842-06-04

**Authors:** 


**CONTENTS OF No. 23.**

Dr. Stewart's account of the Epidemic Influenza prevailing at Paris; known there under the name of "la Grippe," - - - 353
Sketches of American Physicians—Thomas Harris, M. D., - - - 355
BIBLIOGRAPHICAL NOTICE: Dr. Graves on the Diagnosis and Treatment of certain affections of the Heart—Pericarditis, - - 357
EDITORIAL: Western Lancet, - - - - - - - - - - - 359
New York Society for the relief of the Widows and Orphans of Medical men, - - - - - - - - - - 360
Obituary—Sir Chas. Bell, M. Ossan, M. Devergie, M. Fricke, M. J. Fontenelle, - - - - - - - - - - - 360
ANALECTA: On the Cold Water Treatment of Preissnitz at Graeffenberg, 360
Dr. Fisher's Case of Tumour developed in the midst of the Cauda Equina, - - - - - - - - - - 361
Impacted Fracture through the Neck of the Humerus, - - 363
M. Martin on the Preservation of Cantharides, - - - 363
M. Chomel on Acute and Chronic Amygdalitis, - - - 364
Dr. Stokes on Softening of the Heart, &c., - - - - - 366
Dr. Lohmeyer on the results of Revaccination in the Prussian Army, in the year 1840, - - - - - - - - 367

